# Trends in needlestick injury incidence following regulatory change in Ontario, Canada (2004–2012): an observational study

**DOI:** 10.1186/s12913-015-0798-z

**Published:** 2015-04-01

**Authors:** Andrea Chambers, Cameron A Mustard, Jacob Etches

**Affiliations:** Institute for Work and Health, Toronto, Ontario Canada; Dalla Lana School of Public Health, University of Toronto, Toronto, Ontario Canada

**Keywords:** Needlestick, Surveillance, Occupational injury, Infection control, Regulation

## Abstract

**Background:**

A number of jurisdictions have used regulation to promote the adoption of safety-engineered needles as a primary solution to reduce the risk of needlestick injuries among healthcare workers. Regulatory change has not been complemented by ongoing efforts to monitor needlestick injury trends which limits opportunities to evaluate the need for additional investment in this area. The objective of this study was to describe trends in the incidence of needlestick injuries in Ontario prior to and following the establishment of regulation to promote the adoption of safety-engineered needles.

**Methods:**

An observational study of needlestick injuries obtained from two independent administrative data sources (emergency department records for the treatment of work-related disorders and workers' compensation claims) for a population of occupationally-active adults over the period 2004–2012.

**Results:**

Comparing the year prior to the regulation being established (2006) to three years after the regulation came into effect (2011), needlestick injury rates in the health and social services sector that were captured by workers’ compensation claims declined by 31% and by 43% in the work-related emergency department records. Rates of workers’ compensation claims associated with needlestick injuries declined by 31% in the hospital sector, by 67% in the long-term care sector and have increased by approximately 1% in nursing services over the period 2004–2012.

**Conclusions:**

Two independent administrative data sources documented an overall reduction in needlestick injuries in the province of Ontario following a regulatory requirement to adopt safety-engineered needles; however, a substantial burden of occupational needlestick injuries persists in this setting.

## Background

Needlestick injuries represent an important burden of occupational injury in the health care sector. A number of jurisdictions, including the province of Ontario, Canada, turned to regulation to accelerate the adoption of safety-engineered needles (SENs) for the prevention of needlestick injuries. In 2005, 3-years prior to regulation being established in Ontario, a national survey of the work and health of nurses in Canada found that nearly half of surveyed nurses reported being injured by a needle or another sharp tool at some point during their career and 11% reported such an injury in the previous year [1].

Safer needle regulatory standards seek to reduce the burden of needlestick injuries and prevent the potential transmission of blood borne pathogens (e.g., HIV, hepatitis B and C) between patients and healthcare workers. Arguments supporting the need for regulation on needle safety have acknowledged the psychological consequences of the post-exposure experience and the significant cost implications associated with post-exposure testing and treatment [2,3].

Ontario’s regulation on needle safety (474/07) was established under the *Occupational Health and Safety Act* in 2007 [4]. The regulation came into effect for hospitals and psychiatric institutions in September 2008, long-term care homes in April 2009, and all other workplaces in July 2010. In Ontario, the Ministry of Labour sets, communicates and enforces regulation under this Act. Since the regulation was established, and as of 2013–2014, the Ministry of Labour’s annual sector-specific enforcement plans have described a continued focus on compliance with Ontario’s regulation on needle safety.

Regulation on needle safety requires employers to provide workers with SENs: devices that have been engineered to eliminate or minimize the risk of skin puncture injury and that have been licensed as a medical device by Health Canada [4]. This includes the use of needleless devices. Unlike the United States, British Columbia and Alberta, Ontario's regulation on needle safety does not include requirements to replace other sharp medical devices (e.g., suture needles, scalpels, and lancets).

Advocates for safer needle regulation have estimated that the mandatory use of these devices could eliminate up to 90% of injuries [5]. When regulation was being developed in the province, a case study carried out at one Ontario hospital reported an 80% decline in needlestick injuries following a hospital-wide transition to SENs [6]. In terms of the ability of SENs to reduce risk of injury, there is some evidence supporting the efficacy of SENs for the prevention of needlestick injuries; however, needlestick injuries continue to be reported despite the availability of SENs [7]. There is also some evidence to support an association between the degree of user manipulation required to activate a safety device and its ability to prevent needlestick injuries [8]. Safer needle regulation in Ontario provided discretion to individual hospitals concerning the specific type of SEN (passive or semi-automatic) to adopt.

Despite the significant investment associated with a system transition to SENs, there is currently no routine surveillance of needlestick injuries in the province of Ontario. To understand outcomes following regulatory change it is important to monitor progress in terms of impact and implementation. Workers’ compensation claims are frequently used to examine trends in occupational injury burden. There have been concerns about the integrity of reporting to the compensation system overtime, which limits some stakeholders' confidence in reported trends. Emergency department records for the treatment of work-related disorders have been used as an alternative source of information to describe occupational injury trends in the province of Ontario [9]. The objective of this study was to make use of two independent administrative data sources (work-related emergency department records and workers' compensation claims) to describe and compare trends in the rate of needlestick injuries over the period 2004–2012 in Ontario, Canada.

## Methods

### Study design

An observational study of needlestick injuries obtained from two independent administrative data sources (work-related emergency department records and workers' compensation claims) for a population of occupationally active adults in the Canadian province of Ontario over the period 2004–2012.

### Data sources

Administrative records maintained by the Ontario Workplace Safety & Insurance Board partially capture needlestick injuries. A proportion of needlestick injuries that are reported to the employer may lead to a lost-time claim. If the worker experiences lost-time arising from the exposure incident or requires medical treatment (including diagnostic testing or prophylactic treatment), the incident is to be documented in a Worker’s Report of Injury/Disease (Form 6). In addition to these reports, some employers follow a surveillance protocol when a worker is exposed to, or is suspected of having been exposed to an infectious disease through a needlestick injury. These types of incidents are captured in the Workplace Safety and Insurance Board’s Program for Exposure Incident Reporting. The purpose of this voluntary reporting program is to obtain information about the exposure incident should an illness or disease develop in the future. Exposures may be reported either by the employer or the worker by completing the Worker’s Exposure Incident Form (3958A). An aggregate data request to the Workplace Safety and Insurance Board provided counts of needlestick injuries by year (2004–2012), for all rate groups combined and for each of the following rate groups in the health and social services sector: long-term care (851), homes for residential care (852), hospitals (853), nursing services (857), and group homes (858). Over the period 2004–2012 there were 16,364 workers’ compensation claims or exposure incident reports associated with needlestick injuries.

In 2000, Ontario mandated reports of all emergency department visits to the National Ambulatory Care Reporting System. When emergency department visits are determined to be work-related, the responsibility for payment code is assigned to the Workplace Safety and Insurance Board. Work-related emergency department records over the period 2004–2011 were obtained from the Canadian Institute for Health Information who currently maintains the National Ambulatory Care Reporting System. For each record, a main problem and up to 9 other problem codes are assigned using the International Classification of Diseases version 10, Canadian edition (ICD10-CA). Needlestick injuries were defined using the ICD10-CA external cause code for a contact with a hypodermic needle and a series of main problem codes specific to wounds, superficial injuries and other injuries. The external cause code describing contacts with hypodermic needles was only introduced in the fiscal year 2006–2007. Counts of needlestick injuries for the first three months of 2006 were imputed based on the average number of needlestick injuries reported monthly for this year of data in order to examine trends over the period 2006–2011. Over the period 2006–2011 there were 4,325 work-related emergency department records associated with the treatment of needlestick injuries.

Full-time equivalents for the workers compensation claims were based on administrative data from the Workplace Safety and Insurance Board. Employer reported insurable earnings were divided by the average hourly wage for each rate group to estimate the number of hours worked. Full-time equivalents for the work-related emergency department records were based on estimates of annual hours worked from Statistics Canada’s Labour Force survey. To estimate full-time equivalents, the number of hours was divided by 2,000 assuming a person works 2,000 hours per year.

### Analysis

Rates were calculated by dividing the total number of needlestick injuries by the estimate of annual hours worked expressed per 10,000 full-time equivalents. It is important to note that emergency department records do not include a coding scheme to identify specific industries or occupations. Therefore, records of needlestick injuries from this data source include injuries that occur both within and outside the health and social services sector. The ratio of the frequency of compensation claims associated with needlestick injuries in the health and social services to the frequency of claims associated with needlestick injuries in all sectors was used to estimate the number of work-related emergency department records associated with needlesticks in the health and social services. Percent changes in the rates were estimated for two time periods: 2004 vs 2012 and 2006 vs 2011 (the time period available for both data sources). As a result of low counts of needlestick injuries in specific rate groups in the health and social services, rates were only calculated for three rate groups (i.e., long-term care; hospitals, nursing services).

Ethics approval was obtained from the Research Ethics Board at the University of Toronto.

## Results

Table 1 reports the frequency of needlestick injuries captured in compensation claims and work-related emergency department records for all sectors and specifically the health and social services sector over a 9-year period for compensation claims and a 6-year period for work related emergency department records. Needlestick injuries from the health and social services sector represented on average, 63% of all needlestick injury claims over the period 2004–2012. Table 1 also includes incidence rates per 10,000 full-time equivalents and the relative percent decline between 2006 and 2011. There was a high degree of concordance over this time period between data sources for both the health and social services sector and all sectors combined. The relative percent decline in the needlestick injury rate in the health and social services sector between 2006 and 2011 was 31% and 43% among compensation claims and work-related emergency department records, respectively.

**Table 1:** **Comparison of work-related emergency department records and workers’ compensation claims associated with needlestick injuries (NSIs)**

	**2004**	**2005**	**2006**	**2007**	**2008**	**2009**	**2010**	**2011**	**2012**	**% change**
										**2006 vs 2011**
**WSIB, health & social services sector**										
A) NSIs (N)	1373	1260	1249	1181	1165	1159	959	933	1011	
FTEs	305113	310453	318192	324655	334222	342223	340168	344874	360998	
Rate per 10,000 FTEs	45.0	40.6	39.3	36.4	34.9	33.9	28.2	27.1	28.0	−31.0%
**WSIB, all sectors**										
B) NSIs (N)	1975	1937	1946	1898	1910	1824	1603	1536	1635	
FTEs	4248946	4307323	4297172	4299509	4276161	4107249	4164844	4239941	4323254	
Rate per 10,000 FTEs	4.6	4.5	4.5	4.4	4.5	4.4	3.8	3.6	3.8	−20.0%
Ratio of A:B	0.7	0.7	0.64	0.6	0.6	0.6	0.6	0.6	0.6	
**Work-related ED records, health & social services sector**										
NSIs (N)			527	502	484	430	379	361		
FTEs			558229	589540	609035	617463	630810	674658		
Rate per 10,000 FTEs			9.44	8.52	7.95	6.96	6.01	5.35		−43.3%
**Work-related ED records, all sectors**										
NSIs (N)			821	806	794	677	633	594		
FTEs			6116987	6204218	6262973	6055440	6189746	6289157		
Rate per 10,000 FTEs			1.34	1.30	1.27	1.12	1.02	0.94		−29.6%

Note: FTEs, full-time equivalents; ED, emergency department; WSIB, Workplace Safety and Insurance Board.

**Figure 1:**
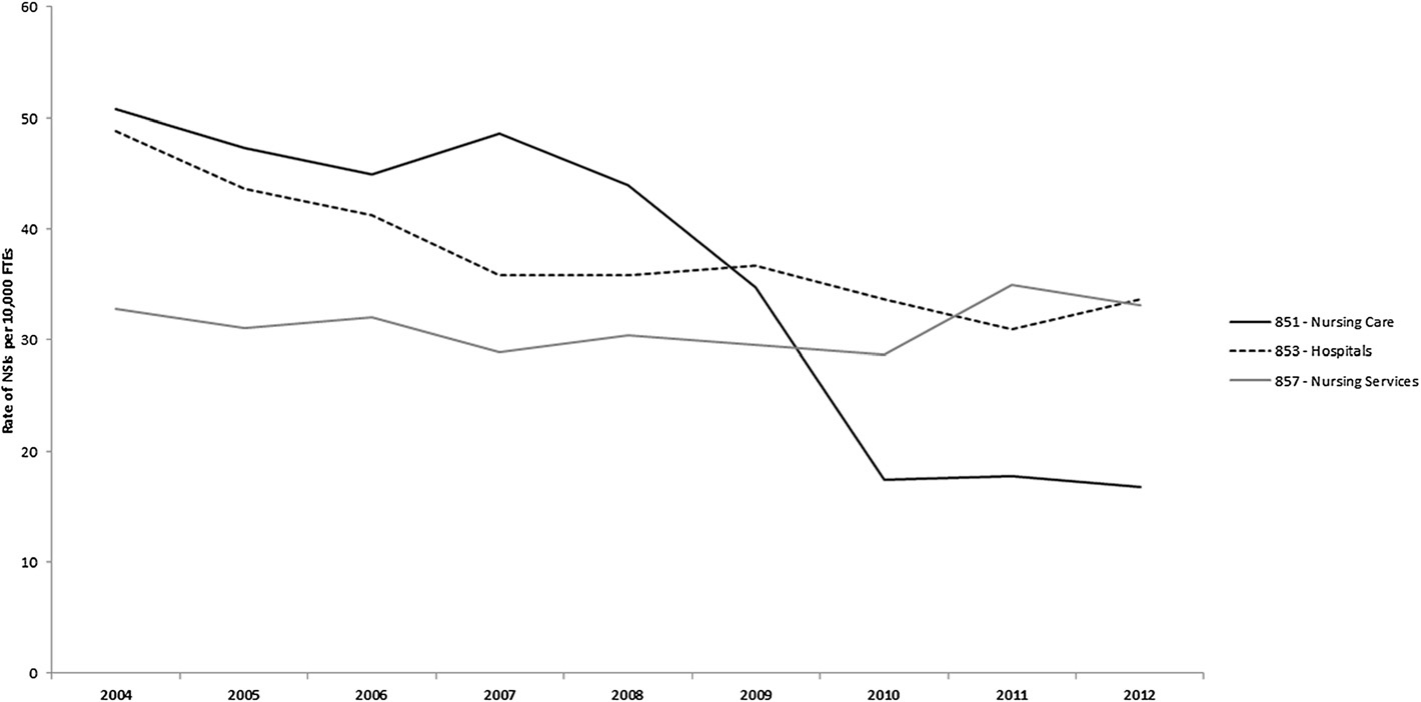
**Rates of workers compensation claims for needlestick injuries (NSI) by sector.**

Figure 1 presents the rate of needlestick injuries captured in compensation claims by three rate groups in the health and social services sector. The relative percent decline over the period 2004–2012 was 31% and 67% in the hospital and long-term care sector, respectively and a 1% increase in the nursing services sector. The ratio of the rate in the hospital sector to the rate in the long-term care sector doubled over the period 2004–2012. The ratio of the rate in the hospital sector to the rate in the nursing services sector decreased by 30% over the period 2004–2012.

## Discussion

This study has documented similar declines in the incidence of needlestick injuries in two independent administrative data sources over the period 2006–2011. While there was some evidence that needlestick injury incidence decreased following a regulatory requirement to adopt SENs, a substantial burden of occupational needlestick injuries persists in the period following the implementation of the regulation.

While trends in the incidence of needlestick injuries were concordant between the two databases, the frequency of claims associated with needlestick injuries was on average 2.5 times higher than the frequency of work-related emergency department records associated with needlestick injuries. This finding is consistent with patterns of care for this type of injury. The majority of these incidents would be managed by occupational health and safety staff. Emergency department services would more likely be used for incidents that occur during off-hours and for those incidents that occur among healthcare workers working in the community setting (e.g., home care).

The gradual decline in needlestick injuries prior to and following regulatory change in Ontario is concordant with trends observed in British Columbia, Canada where regulation was established in 2007 [10]. The United States observed a more immediate drop in the rate of needlestick injuries following the passing of the *Needlestick Safety and Prevention Act* in 2000, which was followed by a plateau [11]. The observation that needlestick injuries did not drop substantially following regulatory change in Ontario is consistent with the timing of the regulation being established. The United States was the first jurisdiction to pass legislation mandating the use of SENs back in 2000 and seven years prior to Ontario establishing regulation. SENs were available on the market for early adopters in Ontario's workplaces for several years prior to regulation being established. The case study published in 2006 that described the successes of one Ontario hospital that voluntarily integrated SENs may have motivated other employers to slowly adopt SENs [6]. Prior to the development of the regulation, inspectors could order employers to implement safety devices citing general requirements under the *Occupational Health and Safety Act* [12]. One of the duties of employers in Ontario under the Act is to “take every precaution reasonable in the circumstances for the protection of a worker” [12]. Inspectors would cite organizations under this section of the Act in specific cases and areas where needlestick injury rates were elevated and where there was insufficient action being taken to reduce risk. One year before the *Safe Needles Save Lives Act* was introduced in the Ontario provincial legislature, it was reported that inspectors visited over 192 healthcare facilities and 68 orders were given for needlestick injuries [13]. The rationale for more specific wording on the mandatory uptake of SENs was rationalized by the amount of requests for appeals and the need to accelerate the uptake of these devices. There was a more substantial reduction following regulatory change in the long-term care sector. This may reflect less proactive adoption of SENs prior to the establishment of the regulation in this sector. While there is evidence to support the efficacy of SENs for the prevention of needlestick injuries [7], a number of other influences that accompany regulatory change such an increased awareness and knowledge could have contributed to the decline in injury rates. The analysis also demonstrated that needlestick injuries continue to occur in the healthcare setting despite the availability of SENs. This study was complemented by a qualitative case study that helped contextualize why needlestick injuries continue to occur [14]. There are a number of product limitations and environmental constraints that explained ongoing needlestick injuries. Despite the increased availability of SENs, needlestick injuries can occur before safety devices are activated or during the activation process [14]. This risk is elevated due to unpredictable patient interactions, distractions, and crowding. Some SENs are perceived to be more awkward to work which can result in staff taking safety caps off the devices or taking non-safety needles from other areas where SENs were not deemed to be clinically appropriate. These limitations help explain why we continue to observe needlestick injuries following a system transition to SENs.

An important strength of this study is the use of two independent administrative data sources to examine trends in the incidence of needlestick injury during a period of regulatory change that promoted the uptake of SENs in the provincial health care system. The results of this study should be interpreted with the following cautions. Not all needlestick injures will require emergency services, result in a lost-time claim, or result in a form being submitted to the Program for Exposure Incident Reporting. As a result, the ascertainment of needlestick injury is incomplete. While the administrative records used in this study have the advantage of describing trends overtime, it is important to acknowledge the potential for changes in reporting behavior over this time period. Increased attention to needlestick injury prevention during the period of regulatory change may have resulted in increased reporting of needlestick injuries. Alternatively, an increase in injury risk may have also occurred as healthcare workers learned to adapt to the new technology. Finally, we acknowledge that the true incidence of needlestick injuries is underestimated in both emergency department records and compensation claims: a number of studies that have examined levels of under-reporting among front-line workers suggest anywhere from 30-90% of needlestick injuries go unreported [15-21]. The need to identify further opportunities to reduce needlestick injuries was emphasized on the occasion of the tenth anniversary of the *Needlestick Safety and Prevention Act* [22]. A Consensus Statement and Call to Action drafted by members of a multi-stakeholder steering committee acknowledged that while substantial progress has been made, preventable sharps injuries and blood exposures continue to occur in healthcare settings. The committee recognized that one cannot assume that all issues will be resolved following the enactment of regulatory standards to promote the uptake of safety-engineered medical sharps and that a renewed commitment was needed to achieve further progress [22].

While there has been a long tradition in the use of regulatory standards to protect the health and safety of workers [23-25], evidence for the effectiveness of regulatory standards has been uneven [26,27]. Controlled studies examining the efficacy of SEN implemented in healthcare have documented substantial declines in needlestick injuries; however, similar declines have not been observed following system uptake of this technology [7].

## Conclusions

In conclusion, we have used two independent administrative data sources to document an overall reduction in needlestick injuries in the province of Ontario following a regulatory requirement to adopt SENs. However, a substantial burden of occupational needlestick injury persists in this setting and further attention is required to identify opportunities to reduce the incidence of occupational needlestick injuries. This study draws attention to the challenge of scaling-up evidence-based interventions for widespread dissemination. It has also demonstrated the importance of continuing to monitor and invest in sustained efforts to prevent needlestick injuries following regulatory change.

## Consent

Please note authorization to use administrative records for research purposes was obtained from the Privacy Offices of the Ontario Workplace Safety and Insurance Board. Analysts at CIHI provided the data requested from the NACRS database following the completion of a Non-Disclosure/Confidentiality Agreement form. All data released by CIHI must comply with their Privacy and Confidentiality of Health Information at CIHI: Principles and Policies for the Protection of Health Information, and Health Facility Identifiable Information Policy.

## Abbreviation

SEN: Safety-engineered needle
